# Aberrant Transforming Growth Factor‐β Activation Recruits Mesenchymal Stem Cells During Prostatic Hyperplasia

**DOI:** 10.5966/sctm.2015-0411

**Published:** 2016-09-07

**Authors:** Long Wang, Liang Xie, Francis Tintani, Hui Xie, Changjun Li, Zhuang Cui, Mei Wan, Xiongbing Zu, Lin Qi, Xu Cao

**Affiliations:** ^1^ Department of Orthopedic Surgery and Institute of Cell Engineering, School of Medicine, Johns Hopkins University, Baltimore, Maryland, USA; ^2^ Department of Urology, Xiangya Hospital, Central South University, Changsha, Hunan, People's Republic of China; ^3^ State Key Laboratory of Oral Diseases, West China Hospital of Stomatology, Sichuan University, Chengdu, Sichuan, People's Republic of China

**Keywords:** Benign prostatic hyperplasia, Transforming growth factor‐β, Mesenchymal stem cells, Stroma, Fibrosis

## Abstract

Benign prostatic hyperplasia (BPH) is the overgrowth of prostate tissues with high prevalence in older men. BPH pathogenesis is not completely understood, but it is believed to be a result of de novo overgrowth of prostatic stroma. In this study, we show that aberrant activation of transforming growth factor‐β (TGF‐β) mobilizes mesenchymal/stromal stem cells (MSCs) in circulating blood, which are recruited for the prostatic stromal hyperplasia. Elevated levels of active TGF‐β were observed in both a phenylephrine‐induced prostatic hyperplasia mouse model and human BPH tissues. Nestin lineage tracing revealed that 39.6% ± 6.3% of fibroblasts and 73.3% ± 4.2% smooth muscle cells were derived from nestin^+^ cells in *Nestin‐Cre*, *Rosa26‐YFP^flox/+^*mice. Nestin^+^ MSCs were increased in the prostatic hyperplasia mice. Our parabiosis experiment demonstrate that nestin^+^ MSCs were mobilized and recruited to the prostatic stroma of wild‐type mice and gave rise to the fibroblasts. Moreover, injection of a TGF‐β neutralizing antibody (1D11) inhibits mobilization of MSCs, their recruitment to the prostatic stroma and hyperplasia. Importantly, knockout of TβRII in nestin^+^ cell lineage ameliorated stromal hyperplasia. Thus, elevated levels of TGF‐β‐induced mobilization and recruitment of MSCs to the reactive stroma resulting in overgrowth of prostate tissues in BPH and, thus, inhibition of TGF‐β activity could be a potential therapy for BPH. Stem Cells Translational Medicine *2017;6:394–404*

**Significance Statement**

Activation of transforming growth factor‐β (TGF‐β) plays a role in the pathogenesis of benign prostatic hyperplasia. TGF‐β activation results in recruitment of MSCs and their subsequent differentiation into prostatic stromal cells. Thus, careful modulation of TGF‐β activity could provide potential therapeutic options in the management of benign prostatic hyperplasia.

## Introduction

Benign prostatic hyperplasia (BPH) is a nonmalignant overgrowth of the prostatic tissue within the transition zone affecting approximately 50% of men aged 51–60 years and 90% by the ninth decade of life 1. BPH is associated lower urinary tract symptoms (LUTS), including storage and voiding symptoms that can progress to urinary retention, bladder dysfunction, and renal impairment if left untreated or treated ineffectively. Despite its prevalence, the exact etiology of BPH remains poorly understood. As a result, current therapies including α‐blockers and 5‐α reductase inhibitors 2 are not completely effective 3. In addition, these treatments could lead to vascular side effects 4 and sexual dysfunction 5. Surgical intervention is reserved for patients who have failed medical therapy or present with moderate‐to‐severe LUTS.

BPH is characterized by the increased stromal compartment of the prostate with age. The stromal‐to‐epithelial ratio is relatively constant at 2:1 from birth to 40 years of age in normal glands increasing to 5:1 in BPH 6. Embryonic reawakening 7, 8, imbalance between androgen and estrogen 9, chronic inflammation 10, 11, stem cell defects 12, and epithelial‐stromal interactions 13, 14 have been implicated in the pathogenesis of BPH. Investigation of the morphological analogies between the fetal prostatic stroma and the BPH stroma nodules showed a similar developmental profile: from immature mesenchyme toward fibroblastic stroma, culminating in predominantly smooth muscle 8. Androgen/androgen receptor (AR) signaling plays an important role in BPH development by influencing the stromal cell paracrine factors that can regulate the adjacent epithelial growth and differentiation 13, 14, 15. Inversely, epithelial cells contribute to the increased stromal cell population via epithelial‐mesenchymal transition (EMT) 16, 17. Therefore, BPH is a stromal enlargement disease likely associated with reawakening of embryonic inductive properties.

High levels of transforming growth factor‐β (TGF‐β) activities are often observed in the pathogenesis of BPH 18. Induction of fibroplasia and collagenous micronodules was observed with TGF‐β1 overexpression in transgenic mice 19. Furthermore, the expression of TGF‐β receptor II protein is associated with increased prostatic gland volume and lymphocyte infiltration in BPH patients 20. Among the three members of the TGF‐β subfamily, TGF‐β1 is predominantly expressed in the prostate 21, 22, 23. TGF‐β is synthesized and deposited into the extracellular matrix (ECM) as a latent complex with mature TGF‐β bound to latency‐associated peptide (LAP). Active TGF‐β can be released from the complex by cleavage of the LAP 24. Temporo‐spatial activation of latent TGF‐β in the matrix recruits stem/progenitor cells for remodeling and repair in various tissues such as bone 25, 26, 27, vasculature 28, 29, and lung 30. Increasing evidence suggests that TGF‐β constitutes integral components in the crosstalk between stem cells and their microenvironment 31.

Mesenchymal/stromal stem cells (MSCs), also known as multipotent stromal cells, have the capacity of self‐renewal and differentiation into various cell types, thereby supporting tissue growth and regeneration 32, 33. Nestin, detected originally in neuronal stem cells, represents one of the class VI intermediate filament protein. Recently, nestin has been used as a marker for bone marrow derived MSCs that have both self‐renewal and multilineage differentiation capacity 28, 34. The existence of MSCs in prostate has been verified in different studies 12, 35, 36. In the present study, we used several animal models including phenylephrine (PE)‐induced prostatic hyperplasia mice, a parabiosis mice model by *Nestin‐Cre*, *Rosa26‐YFP^flox/+^*mice surgically sutured with wild‐type littermates and conditional knockout *Nestin‐Cre-ER*, *Tgfbr2^flox/flox^* mice to investigate whether nestin^+^ MSCs are mobilized in peripheral circulation, recruited to the prostatic stroma and give rise to the fibroblasts during prostatic hyperplasia. We found that systemic blockade of TGF‐β activity or conditional deletion of the TGF‐β receptor II (*Tgfbr2*) in nestin^+^ cell lineage attenuated stromal hyperplasia with potential as an effective therapy for human BPH.

## Materials and Methods

### Human Specimens

BPH tissue specimens were collected from 32 patients with BPH that were undergoing transurethral resection of the prostate. As controls, normal prostate tissues were obtained from 18 bladder tumor patients younger than 40 undergoing radical cystoprostatectomy. No other inclusion or exclusion criteria were used other than tissue quality after thawing. Our study was performed according to the ethical standards of the 1975 Declaration of Helsinki, as revised in 2008, and was approved by the Central South University Ethic Committee. Informed consent was obtained from all the participating patients.

### Mice Generation

C57BL/6J (wild‐type) mice were purchased from Charles River (Wilmington, MA, http://www.criver.com). *Nestin‐GFP* mouse strain was obtained from Grigori Enikolopov (Cold Spring Harbor Laboratory, Cold Spring Harbor, NY, http://www.cshl.edu). *Nestin‐Cre* and *Rosa26‐YFP* mouse strains were purchased from Jackson Laboratory. *Nestin‐Cre-ER* and *Tgfbr2^flox/flox^* mice were obtained from our previous study 26.

Phenylephrine induction of ventral prostate hyperplasia procedures were performed on 6‐week‐old mice with the methods previously described by Marinese et al. 37. As the ventral lobe is often considered as the common site for prostatic hyperplasia, the ventral prostates were harvested and analyzed. For the time course experiments, PE‐induced mice and the saline‐treated controls were sacrificed at 0, 1, 2, or 4 weeks after initial injection. For the treatment with TGF‐β neutralizing antibody 1D11 (5 mg/kg^−1^; Sanofi Genzyme, Cambridge, MA, https://www.sanofigenzyme.com) or the equivalent dose of control antibody 13C4 were injected intraperitoneally three times per week starting with PE injection.

To generate *Nestin‐Cre-ER*, *Tgfbr2^flox/flox^* (*Tgfbr2* ^−/−^) mice, we crossed hemizygous *Nestin‐Cre-ER* mice with *Tgfbr2^flox/flox^* mice to generate heterozygous *Tgfbr2^flox^* offspring with or without a *Cre* allele. We then intercrossed these offspring to generate the following offspring: *Nestin‐Cre-ER*, *Tgfbr2^flox/flox^* (conditional knockout mice referred as *Tgfbr2* ^−/−^); *Nestin‐Cre-ER* (referred as *Tgfbr2^+/+^*); *Nestin‐Cre-ER*, *Tgfbr2^flox/+^* (heterozygous conditional knockout mice, referred to as *Tgfbr2* ^+/−^); and mice without *Nestin‐Cre-ER*. Inducible gene deletion was performed by tamoxifen (dissolved in corn oil, 75 mg/kg^−1^) intraperitoneal injections every other day starting with PE injection, and the *Nestin‐Cre-ER*, *Tgfbr2^flox/flox^* mice were euthanized 28 days after initial injection.

*Nestin‐Cre* mice were crossed with *Rosa26‐YFP* reporter mice to generate the following offspring: *Nestin‐Cre*, *Rosa26‐YFP^flox/+^* (mice expressing YFP in nestin lineage cells) and mice without *Nestin‐Cre*.

We determined the genotype of the mice by polymerase chain reaction analyses of genomic DNA isolated from mouse tails using the following primer sequences:

*Nestin‐GFP* forward, 5′-GGAGCTGCACACAACCCATTGCC-3′ and reverse 5′-GATCACTCTCGGCATGGACGAGC-3′.
*Nestin‐Cre* forward, 5′‐GCGGTCTGGCAGTAAAAACTATC -3′ and reverse, 5′-GTGAAACAGCATTGCTGTCACTT-3′.
*Rosa26-YFP* allele mutant, 5′-AAGACCGCGAAGAGTTTGTC-3′ and common, 5′-AAAGTCGCTCTGAGTTGTTAT-3′
and wild‐type, 5′-GGAGCGGGAGAAATGGATATG-3′, product size 600 bp for wild‐type and 320 bp for mutant;
*Nestin‐Cre-ER* forward 5′-CAAATAGCCCTGGCAGAT-3′ and reverse 5′-TGATACAAGGGACATCTTCC-3′.
*loxP Tgfbr2* allele forward, 5′-TAAACAAGGTCCGGAGCCCA-3′ and reverse 5′-ACTTCTGCAAGAGGTCCCCT-3′.

No statistical method was used to predetermine sample size. Male mice were randomly assigned to both control and testing groups, each typically containing three to five animals.

### Parabiosis

Four‐week‐old male mice were paired as described previously 29, 38. A 4‐week‐old *Nestin‐Cre*, *Rosa26‐YFP^flox/+^*or *Nestin‐Cre* (control) mouse was surgically joined to a wild‐type littermate. Briefly, the mice were anesthetized, and longitudinal skin incisions were performed from the elbow to the knee joint of each mouse. The elbow and knee joints were attached by a surgical suture, and then the dorsal and ventral skin was stitched by a continuous 5‐0 Vicryl (Ethicon, Somerville, NJ, http://www.ethicon.com) suture. Each parabiotic pair was housed in a clean cage with moistened food pellets on the floor to decrease the movement of reaching for food while adjusting to parabiotic existence. After 2 weeks, shared blood circulation between the mice was confirmed by injection of Evans blue dye (Sigma‐Aldrich, St. Louis, MO, https://www.sigmaaldrich.com). The parabiotic wild‐type partner was injected with either PE (10 mg/kg^−1^/day^−1^) or saline for 4 weeks before the mice were sacrificed. Ventral prostates from the mouse of each parabiotic pair were harvested for analysis.

All animals were maintained in the Animal Facility of the Johns Hopkins University School of Medicine. The experimental protocols for both species were reviewed and approved by the Institutional Animal Care and Use Committee of the Johns Hopkins University.

### Enzyme‐Linked Immunosorbent Assay of TGF‐β1 in Plasma

We detected the concentration of active TGF‐β1 in plasma of mice with an enzyme‐linked immunosorbent assay (ELISA) development kit (R&D Systems, Minneapolis, MN, https://www.rndsystems.com) according to the manufacturer's instructions.

### Immunohistochemistry, Immunohistofluorescence, and Histomorphometry

We collected and fixed the ventral prostate in 10% buffered formalin overnight at 4°C and embedded them in paraffin or optimal cutting temperature compound (Sakura Finetek, Torrance, CA, http://www.sakura-americas.com). Five‐micrometer‐thick sections of the prostate were processed for hematoxylin and eosin (H&E) staining. Masson's trichrome staining was performed using a standard protocol (HT15‐1KT and HT1079; Sigma‐Aldrich). The procedure for immunostaining was described previously 26. Briefly, we incubated sections with primary antibodies against pSmad2/3 (1:50, sc‐11769; Santa Cruz Biotechnology, Santa Cruz, CA, http://www.scbt.com), green fluorescent protein (GFP; 1:500, 600‐101‐215; Rockland, Limerick, PA, http://www.rockland-inc.com), α‐smooth muscle actin (α‐SMA; 1:200, ab5694; Abcam, Cambridge, U.K., http://www.abcam.com), vimentin (1:200, ab92547; Abcam), Sca1 (1:100, ab51317; Abcam), and CD90 (1:100, ab3105; Abcam) overnight at 4°C. For immunohistochemical staining, biotinylated secondary antibodies and a horseradish peroxidase‐streptavidin detection system (Dako, Carpinteria, CA, http://www.dako.com) was used for chromatic visualization. For immunofluorescent staining, secondary antibodies conjugated with fluorescence were added, and slides were incubated at room temperature for 1 hour while avoiding light. Histological images were taken with an Olympus DP71 microscope, and quantitative analysis was carried out in a blinded fashion using Image pro plus 6.0 (Media Cybernetics, Inc., Rockville, MD, http://www.mediacy.com).

### Flow Cytometry Analysis

For the analysis or sorting of GFP^+^CD45^−^ or CD105^+^CD90^+^CD45^−^CD11b^−^ cells in peripheral circulation, blood samples were collected from mice by intracardiac puncture. After the process of red blood cell lysis with commercial ammonium‐chloride‐potassium lysis buffer (Quality Biological, Gaithersburg, MD, http://www.qualitybiological.com), cells were washed and counted. We incubated equal numbers of cells for 45 minutes at 4°C with fluorescein isothiocyanate (FITC)‐conjugated anti‐GFP (PA1‐46326; Thermo Fisher Scientific, Waltham, MA, https://www.thermofisher.com) and peridinin chlorophyll protein (perCP)‐conjugated anti‐CD45 (103132; BioLegend, San Diego, CA, http://www.biolegend.com). We also incubated cells with phycoerythrin (PE)‐conjugated anti‐Sca1 (108108; BioLegend), allophycocyanin‐conjugated anti‐CD90 (140311; BioLegend), perCP‐conjugated anti‐CD45 (103132, BioLegend), and FITC‐conjugated anti‐CD11b (101208, BioLegend) antibodies. Probes were analyzed using Becton Dickinson FACSCalibur flow cytometer (Becton Dickinson, Franklin Lakes, NJ, http://www.bd.com) and CellQuest software.

### Isolation and Culture of Murine GFP‐Labeled Prostatic Nestin^+^ Cells

To obtain prostatic nestin^+^ cells, ventral prostate isolated from 6‐week‐old male Nestin‐GFP transgenic mice after euthanization were carefully dissected under sterile conditions. Subsequently, the procured tissues were submerged in 5% low melting point agarose (Thermo Fisher) for protection from digestion. After solidification of the agarose, prostatic cells were isolated by collagenase in α‐minimal essential medium (α‐MEM; Corning Cellgro, Manassas, VA, http://www.cellgro.com). The digestion was stopped by addition of an equal volume of fetal bovine serum (FBS; Atlanta Biologicals, Flowery Branch, GA, https://www.atlantabio.com). The cell suspension was passed through a 70‐µm nylon cell strainer (BD Biosciences, San Jose, CA, http://www.bdbiosciences.com), washed three times with α‐MEM. Cells aliquots were incubated for 60 minutes at 4°C with FITC‐conjugated GFP antibody and perCP‐conjugated CD45 antibody. Acquisition was performed on a fluorescence‐activated cell sorting (FACS) Aria model (BD Biosciences), and analysis was performed with a FACS DIVE software version 6.1.3 (BD Biosciences). To isolate individual clonal strains, the sorted GFP^+^CD45^–^ cells were passed consecutively through 16‐ and 20‐gauge needles to obtain single cell suspensions. For single colony forming, 1 × 10^3^ cells were cultured in 100‐mm dishes with α‐MEM containing penicillin (100 U/ml; Sigma‐Aldrich), streptomycin sulfate (100 μg/ml; Sigma‐Aldrich), and 20% FBS at 37°C in a 5% CO_2_ humidified incubator. Individual, well‐separated colonies were selected with cloning cylinders and the number of cells was individually expanded by passaging and confirmed by flow cytometry.

### Fibrogenic, Osteogenic, Adipogenic, and Chondrogenic Differentiation

For fibrogenic differentiation, cells were seeded at a density of 5 × 10^3^/cm^−2^ with α‐MEM supplemented with 10% FBS, 100 ng/ml^−1^ connective tissue growth factor (BioVendor, Asheville, NC, https://www.biovendor.com), and 50 μg/ml^−1^ ascorbic acids (Sigma‐Aldrich), as described previously 39. Cultures in α‐MEM supplemented with 10% FBS served as a negative control. After 3 weeks of differentiation, the fibroblastic differentiation of the cells was evaluated by vimentin staining.

For osteogenic differentiation, cells were seeded at a density of 5 × 10^3^/cm^−2^ with α‐MEM supplemented with 10% FBS, 10^−7^ M dexamethasone (Sigma‐Aldrich), 10 mM β‐glycerol phosphate (Sigma‐Aldrich), and 50 μM ascorbate‐2‐phosphate (Sigma‐Aldrich). Cultures in α‐MEM supplemented with 10% FBS served as a negative control. After 3 weeks of differentiation, the mineralization capacity of the cells was evaluated by Alizarin Red S staining (2% of Alizarin Red S [Sigma‐Aldrich] dissolved in distilled water with the pH adjusted to 4.2).

For adipogenic differentiation, cells were seeded at a density of 1 × 10^4^/cm^−2^ with α‐MEM supplemented with 10% FBS, 10^−6^ M dexamethasone, 0.5 μM IBMX (Sigma‐Aldrich), and 10 ng/ml insulin (Sigma‐Aldrich) for 2 weeks. Cultures of cells in α‐MEM supplemented with 10% FBS served as a negative control. Lipid accumulation was identified by Oil Red O staining (0.5 g of Oil Red O [Sigma‐Aldrich] was dissolved in 100 ml of isopropanol [Sigma‐Aldrich] and diluted to 60% with distilled water).

For chondrogenic differentiation, cells were seeded in polypropylene tubes (5 × 10^5^ per tube^−1^) with high‐glucose Dulbecco's modified Eagle's medium (DMEM; Corning Cellgro) supplemented with 10^−7^ M dexamethasone, 1% ITS (Sigma‐Aldrich), 50 μM ascorbate‐2‐phosphate, 1 mM sodium pyruvate (Sigma‐Aldrich), 50 μg/ml of proline (Sigma‐Aldrich), and 20 ng/ml^−1^ of TGF‐β3 (R&D Systems). Culture cells in high‐glucose DMEM supplemented with 10% FBS served as a negative control. After 3 weeks in culture, the pellets were fixed in 10% buffered formalin for 24 hours and embedded in paraffin. Then 4‐mm thick sections were processed for toluidine blue staining (1 g of toluidine blue [Sigma‐Aldrich] was dissolved in 100 ml of 70% alcohol and diluted to 10% with 1% sodium chloride, pH adjusted to 2.3).

### Statistical Analysis

Data are presented as mean ± SD and were analyzed using two‐tailed Student's *t* tests for comparisons between two groups, one‐way analysis of variance (ANOVA) with Bonferroni post hoc test for multiple comparisons. For all experiments, *p* < .05 was considered to be significant and was indicated by a single asterisk; *p* < .01 was indicated by a double asterisk. No statistical method was used to predetermine the sample size. The experiments were randomized. The investigators were not blinded to allocation during experiments and outcome assessment.

## Results

### Elevated TGF‐β Activity in Prostatic Stromal Hyperplasia

**Figure 1:**
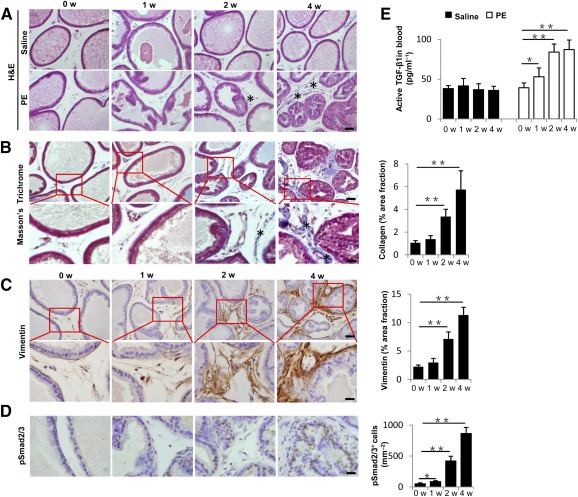
TGF‐β signaling is activated during prostatic stromal hyperplasia in PE‐induced mice. **(A):** H&E staining of ventral prostate sections from either saline‐ or PE‐treated mice at 0, 1, 2, and 4 weeks after the initial injection. The asterisk (∗) marks the reactive stroma. Scale bars = 50 μm. **(B):** Masson's trichrome staining of ventral prostate sections; blue colors indicate collagen fibers (∗). Bar graph on the right shows the quantitative analyses of the percentage of collagen stained areas. Panels in the bottom are higher magnification of red boxed area in the top panels. Scale bars = 50 μm in top panels, 20 μm in bottom panels. **(C):** Immunohistochemical (left) and quantitative (right) analyses of vimentin (brown) at 0, 1, 2, and 4 weeks after initial PE injection. Panels in the bottom are higher magnification of red boxed area in the top panels. Scale bars = 50 μm in top panels, 20 μm in bottom panels. **(D):** Immunohistochemical (left) and quantitative (right) analyses of pSmad2/3 (brown). Scale bar = 20μm. **(E):** Active TGF‐β1 level in peripheral blood of PE‐induced mice versus control mice measured by enzyme‐linked immunosorbent assay. *n* = 5 per group. Data are shown as the mean ± SD. ∗, *p* < .05, ∗∗, *p* < .01 determined by multifactorial analysis of variance. Abbreviations: H&E, hematoxylin and eosin; PE, phenylephrine; TGF, transforming growth factor; w, weeks.

To delineate the potential role of TGF‐β signaling in prostatic stromal hyperplasia, we generated a PE‐induced mouse model 37, 40. Mice induced with PE showed stromal compartment hyperplasia with concomitant papillary in folding and/or piling up of epithelial cells after 4 weeks of injection relative to saline‐treated mice (Fig. 1A). Collagen deposition and stromal content were significantly increased from 2 to 4 weeks after initial PE injection with Masson's trichrome staining (Fig. 1B). In parallel, the number of vimentin^+^ fibroblasts were also increased (Fig. 1C). Ventral prostate sections were immunostained for phosphorylated Smad2/3 (pSmad2/3) and the results demonstrated that pSmad2/3levels were elevated in the nuclei of both epithelial and stromal cells continuously from1week after initial PE injection (Fig. 1D). To examine whether active TGF‐β level in circulation is changed, we measured the concentration of active TGF‐β1 in blood. In PE‐induced mice, active TGF‐β1was significantly higher at 1 week and peaked at 4 weeks relative to the level of week 0 (Fig. 1E). These results indicate that TGF‐β signaling was elevated with reactive stromal component in PE‐induced mice.

**Figure 2:**
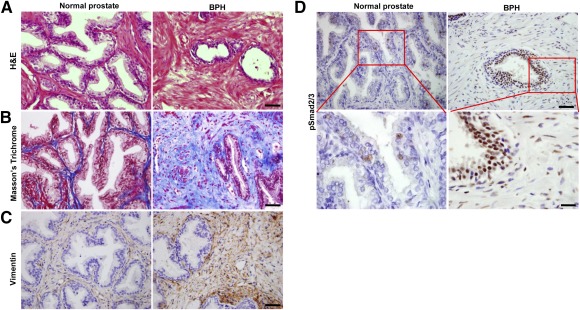
TGF‐β signaling is activated in prostate tissue from BPH patients. **(A):** H&E staining of normal human prostate (left) and BPH tissues (right). Scale bars = 50 μm. **(B):** Masson's trichrome staining indicates collagen deposition (blue) and smooth muscle cells (red). Scale bars = 50 μm. **(C):** Immunostaining of vimentin (brown). Scale bars = 50 μm. **(D):** Immunostaining of pSmad2/3 (brown). Panels in the bottom are higher magnification of red boxed area in the top panels. Scale bars = 50 μm in top panels, 20 μm in bottom panels. Abbreviations: BPH, benign prostatic hyperplasia; H&E, hematoxylin and eosin; TGF, transforming growth factor.

We then examined whether high level of active TGF‐β is directly associated with prostate stroma augmentation in human BPH tissues. A constant increase of the relative and absolute amount of stromal component was observed in all human BPH specimens stained by H&E (Fig. 2A). Masson's trichrome staining showed far more stromal compartment with large quantity of blue‐staining collagen fibers mixed with red‐staining smooth muscle cells in BPH relative to normal prostate tissue (Fig. 2B). Vimentin^+^ area was also more extensive in BPH (Fig. 2C). Again, pSmad2/3^+^ cells were dramatically increased in both the epithelium and stromal cells in BPH samples, whereas fewer pSmad2/3^+^ cells were loosely scattered in normal prostate (Fig. 2D). Thus, high levels of TGF‐β activity are associated with stromal expansion in the pathogenesis of human BPH.

### MSCs Are Recruited to the Stroma During Prostatic Hyperplasia

**Figure 3:**
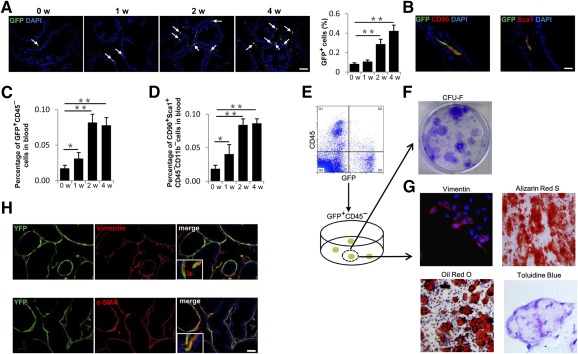
Increased nestin^+^ cells in prostate stroma are a subset of mesenchymal/stromal stem cells. **(A):** Immunofluorescence analysis of prostate tissue sections from Nestin‐GFP transgenic mice at 0, 1, 2, and 4 weeks after initial PE injection using antibody against GFP. Arrows indicate GFP^+^ cells. Bar graph on the right shows the quantitative analyses of the ratio of GFP^+^ cells to total cells. Scale bar = 50μm. **(B):** Double‐immunohistofluorescence analysis of prostate tissue sections from Nestin‐GFP mice using antibodies against GFP and CD90 or Sca1. Scale bar = 20 μm. **(C):** Flow cytometry analysis of GFP^+^CD45^–^cells (percentage of total cells counted) in blood of Nestin‐GFP mice at indicated time points after PE injection. **(D):** Flow cytometry analysis of CD90^+^Sca1^+^CD11b^–^CD45^–^ cells (percentage of total cells counted) in blood of wild‐type mice at indicated time points after PE injection. **(E):** Fluorescence‐activated cell sorting of GFP^+^CD45^−^cells from ventral prostate tissue of 6‐week‐old Nestin‐GFP mice. **(F):** Single CFU‐F‐derived GFP^+^CD45^−^ cell strains were isolated and individually expanded for characterization assays. **(G):** Single GFP^+^CD45^−^ clones were isolated, expanded, and cultured. Cells were separately incubated with fibrogenic media, osteogenic media, adipogenic media, and chondrogenic media, and they were stained for vimentin (upper left panel), Alizarin Red S (upper right panel), Oil Red O (lower left panel), and Toluidine Blue (lower right panel), respectively. **(H):** Double‐immunohistofluorescence analysis of prostate tissue sections from 6‐week‐old *Nestin‐Cre*, *Rosa26‐YFP^flox/+^* mice using antibodies against YFP (green) and vimentin (red, top panels) or α‐SMA (red, bottom panels). Scale bar = 50μm. *n* = 4–5 per group. Data are shown as the mean ± SD. ∗, *p* < .05, ∗∗, *p* < .01 determined by multifactorial analysis of variance. Abbreviations: CFU‐F, colony‐forming unit fibroblast; DAPI, 4′,6‐diamidino‐2‐phenylindole; GFP, green fluorescent protein; PE, phenylephrine; α‐SMA, α‐smooth muscle actin; w, weeks.

To investigate whether high levels of active TGF‐β are involved in the recruitment of MSCs during prostatic hyperplasia, we used Nestin‐GFP transgenic mouse in which nestin^+^ cells were labeled with GFP. GFP^+^ cells increased significantly with time in the prostate stroma of the PE‐induced mice (Fig. 3A). Ventral prostate sections were immunostained with antibodies against CD90 and Sca1, markers for MSCs 41. CD90 and Sca1 were colocalized with GFP in the stroma (Fig. 3B), indicating increase of MSCs. Furthermore, the percentage of GFP^+^CD45^−^ cells in blood circulation were almost twofold at 1 week and fourfold at 2 and 4 weeks after injection of PE relative to baseline at week 0 (Fig. 3C). Similarly, in wild‐type mice, the percentages of CD90^+^Sca1^+^CD45^−^CD11b^−^ cells in blood were also increased with PE injection (Fig. 3D).

To examine whether the recruited GFP^+^ cells in prostate stroma are multipotential MSCs, cells isolated from ventral prostate of Nestin‐GFP mice were sorted by GFP^+^ and CD45‐negative fluorescence (Fig. 3E). The sorted GFP^+^CD45^–^ cells were assessed by colony‐forming unit fibroblast (CFU‐F) assay (Fig. 3F) and displayed CFU‐F activity. Their multipotential was tested for fibrogenesis, osteogenesis, adipogenesis, and chondrogenesis (Fig. 3G). These results suggest that MSCs were mobilized into the peripheral blood and recruited to the stroma during prostatic hyperplasia.

To determine whether prostate stromal cells are differentiated from nestin^+^ cells, we performed nestin‐lineage tracing experiment by crossing *Nestin‐Cre* mice with *Rosa26‐YFP* mice. Both nestin^+^ cells and their descendants were labeled with YFP. In 6‐week‐old *Nestin‐Cre*, *Rosa26‐YFP^flox/+^*mice, double immunostaining of YFP and vimentin or α‐SMA demonstrated that 39.6% ± 6.3% of fibroblasts were derived from nestin^+^ cells, and, 73.3% ± 4.2% smooth muscle cells were of YFP^+^ nestin origin (Fig. 3H). This shows that nestin^+^ cells are an important source of prostate stromal cells.

### Circulating Nestin^+^ Cells Recruited to the Reactive Stroma Give Rise to Fibroblasts

**Figure 4:**
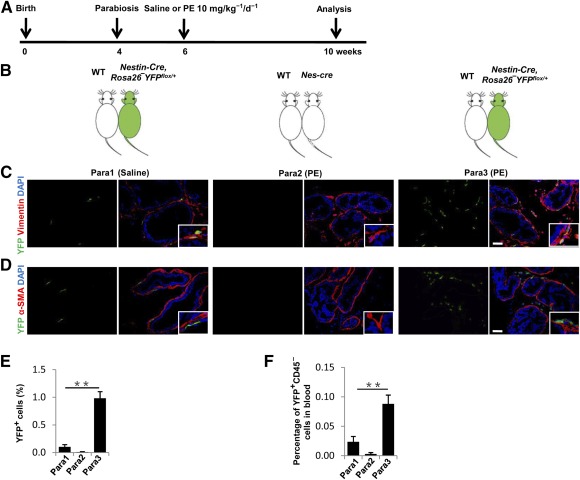
Parabiotic partner‐derived nestin^+^ cells predominantly give rise to fibroblasts in prostate stroma. **(A, B):** Schematic showing the experimental strategy **(A)** and parabiotic pairings setting **(B)**. **(C):** Double‐immunohistofluorescence analysis of prostate tissue sections from wild‐type mice that were linked parabiotically to *Nestin‐Cre* or *Nestin‐Cre*, *Rosa26‐YFP^flox/+^*mice with saline‐ or PE‐treatment using antibodies against YFP (green) and vimentin (red). Scale bar = 50 μm. **(D):** Double‐immunohistofluorescence analysis of prostate tissue sections from wild‐type mice that were linked parabiotically to *Nestin‐Cre* or *Nestin‐Cre*, *Rosa26‐YFP^flox/+^*mice with saline‐ or PE‐treatment using antibodies against YFP (green) and α‐SMA (red). Scale bar = 50 μm. **(E):** Quantitative analyses of the ratio of YFP^+^ cells to total cells in prostate tissue sections. **(F):** Flow cytometry analysis of YFP^+^CD45^–^ (percentage of total cells counted) in blood of wild‐type mouse partners. *n* = 4 per group. Data are shown as the mean ± SD. ∗∗, *p* < .01 compared with the Para1 group. Statistical significance was determined by multifactorial analysis of variance. Abbreviations: DAPI, 4′,6‐diamidino‐2‐phenylindole; PE, phenylephrine; α‐SMA, α‐smooth muscle actin.

To validate that recruited nestin^+^ cells from peripheral blood are involved in prostatic hyperplasia, we generated parabiotic pairs composed of *Nestin‐Cre*, *Rosa26‐YFP^flox/+^* mice and wild‐type littermates (Para1 and Para3). The mice were treated with PE or vehicle after parabiosis for 4 weeks as shown in Figure 4A and 4B. In parallel, *Nes‐Cre* mice were joined to their wild‐type littermates (Para2) as controls of the nonspecific tissue autofluorescence. Notably, YFP^+^ cells, which are descendants of nestin^+^ cells derived from *Nestin‐Cre*, *Rosa26‐YFP^flox/+^*mouse partners through circulation, were detected in the prostate stroma of wild‐type partners in Para3 with PE induction, but they were significantly less in Para1 wild‐type partners (Fig. 4C, 4D). Double‐immunostaining of YFP and vimentin or α‐SMA showed that all of the nestin‐lineage cells were vimentin^+^ but not α‐SMA^+^ (Fig. 4C). YFP^+^CD45^−^ cells in circulating blood were significantly elevated after injection of PE in Para3 relative to Para1 and Para2 control groups (Fig. 4E, 4F). Thus, nestin^+^ cells were mobilized in blood circulation and recruited to the stroma in prostatic hyperplasia.

### Inhibition of TGF‐β Signaling Attenuates Prostatic Stromal Hyperplasia

**Figure 5:**
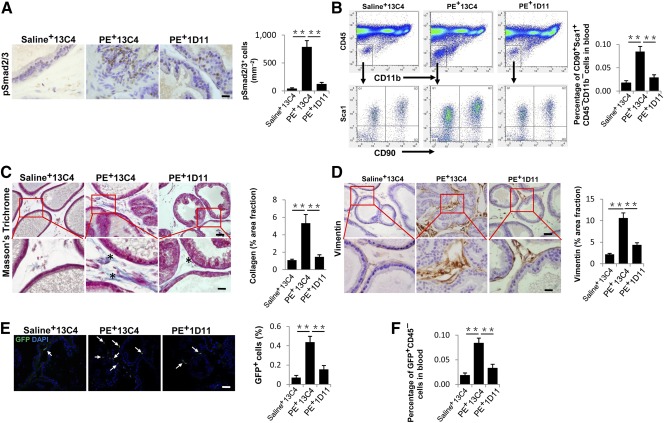
TGF‐β neutralizing antibody (1D11) treatment inhibits prostatic stromal hyperplasia and mesenchymal/stromal stem cell accumulation in PE‐induced mice. Saline or PE‐challenged mice were treated with TGF‐β neutralizing antibody 1D11 (5 mg/kg^−1^) or the equivalent volume of control antibody 13C4 three times per week. **(A):** Immunohistochemical staining (left) and quantitative analyses (right) of pSmad2/3^+^ cells (brown) in prostate tissue of three treatment groups. Scale bar = 20 μm. **(B):** Flow cytometry analysis of the percentage of CD90^+^Sca1^+^CD11b^–^CD45^–^ cells in blood cells of wild‐type mice in three groups. **(C):** Masson's trichrome staining of the prostate tissue of three groups. Quantitative analyses of the percentage of collagen stained areas (asterisk) are shown in the right bar graph. Scale bars = 50 μm in top panels, 20 μm in bottom panels. **(D):** Immunohistochemical staining (left) and quantitative analyses (right) of vimentin (brown). Panels in the bottom are at higher magnification of red boxed area in the top panel. Scale bars = 50 μm in top panels, 20 μm in bottom panel. **(E):** Immunofluorescence staining of GFP (arrows) of Nestin‐GFP mice in three groups. Bar graph shows the quantitative analyses of the ratio of GFP^+^ cells to total cells. Scale bar = 50 μm. **(F):** Flow cytometry analysis of the percentage of GFP^+^CD45^−^ in blood cells of Nestin‐GFP mice in three groups. All data were obtained from saline or PE‐treated mice 4 weeks after initial injection. *n* = 5 per group **(A–E).** Data are shown as the mean ± SD. ∗∗, *p* < .01 determined by multifactorial analysis of variance. Abbreviations: DAPI, 4′,6‐diamidino‐2‐phenylindole; GFP, green fluorescent protein; PE, phenylephrine; TGF, transforming growth factor.

To validate that aberrant activation of TGF‐β in the prostatic stroma mobilizes nestin^+^ cells, we injected the PE‐induced mice intraperitoneally with TGF‐β neutralizing antibody (1D11) or an isotype‐matched murine IgG1 control antibody (13C4). Immunohistological staining showed that injection of TGF‐β antibody 1D11 reduced Smad2/3^+^ phosphorylation in the stroma (Fig. 5A). The percentage of CD90^+^Sca1^+^CD45^−^CD11b^−^ MSCs in blood circulation was also decreased (Fig. 5B). Significant reduction of the collagen accumulation and decrease of vimentin^+^ fibroblasts were observed in the 1D11‐treated mice relative to the mice injected with 13C4 control antibody (Fig. 5C, 5D). Moreover, 4 weeks of 1D11 antibody treatment not only significantly reduced GFP^+^ cells in the prostate stroma, but also reversed the elevated GFP^+^CD45^−^ cells in peripheral blood of the PE‐induced Nestin‐GFP mice (Fig. 5E, 5F). Taken together, these results demonstrate that inhibition of TGF‐β activity could prevent prostate stroma expansion by reducing recruitment of MSCs.

### Knockout of *Tgfbr2* in Nestin^+^ Cells Ameliorates Prostatic Stromal Hyperplasia

**Figure 6:**
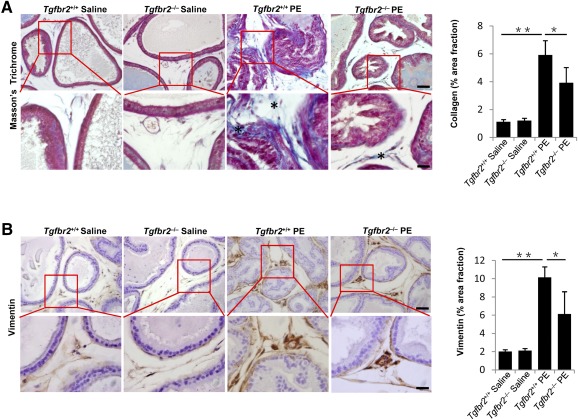
Inducible knockout of *Tgfbr2* in nestin+ cells leads to inhibit prostatic stromal hyperplasia. *Nestin‐Cre-ER*, *Tgfbr2^flox/flox^* mice (*Tgfbr2^−/−^*) and control mice (*Tgfbr2^+/+^*) treated with saline or PE for 4 weeks. **(A):** Masson's trichrome staining of the prostate tissue from the mice. Panels in the bottom are at higher magnification of red boxed area in the top panels. Bar graph on the right shows the quantitative analyses of the collagen (∗). Scale bars = 50 μm in top panels, 20 μm in bottom panels. **(B):** Immunohistochemical staining and quantitative analyses (right) of vimentin (brown). Scale bars = 50 μm in top panels, 20 μm in bottom panels. *n* = 5–6 per group. Data are shown as the mean ± SD. ∗, *p* < .05, ∗∗, *p* < .01 determined by multifactorial analysis of variance. Abbreviation: PE, phenylephrine.

To further demonstrate that local excessive activation of TGF‐β recruits nestin^+^ cells to prostate stroma during pathogenesis of prostatic hyperplasia, we generated *Nestin‐Cre-ER*, *Tgfbr2^flox/flox^* mice by crossing *Nestin‐Cre-ER* with *Tgfbr2^flox/flox^*. *Tgfbr2*was deleted in the nestin^+^ cells by intraperitoneal injection of tamoxifen to the *Nestin‐Cre-ER*, *Tgfbr2^flox/flox^* mice every other day starting with PE injection. Immunostaining of prostatic tissue sections showed that collagen deposition (Fig. 6A) and fibroblasts accumulation (Fig. 6B) in the stroma of *Tgfbr2* ^−/−^ mice were significantly reduced relative to their wild‐type littermates (*Tgfbr2* ^+/+^). These *Tgfbr2* knockout data confirmed that increased TGF‐β activity in the stroma enhances recruitment of nestin^+^ cells for prostatic hyperplasia.

## Discussion

The embryonic reawakening theory implicated a potential role of MSCs in the pathogenesis of BPH. Evidence has recently emerged to support this argument 12, 35, 42, 43, 44. Lin et al. 12 identified and isolated prostatic stromal cells from BPH specimens, which possessed strong proliferative potential and the ability to differentiate to myogenic, adipogenic, and osteogenic lineage. Ceder et al. 35 found a stromal stem/progenitor cells population in both fresh and cultured human prostate tissue. This stromal population was positive for KIT, vimentin, CD133, and SCF, and negative for AC133 and Bcl‐2. Brennen et al. 45 also demonstrated that the number of MSCs constitute 0.01%–1.1% of the total cells in human prostatectomy specimens. These cells expressed CD90, CD73, and CD105 in the absence of hematopoietic lineage markers using flow cytometry assay. However, studies of the physiological or pathological function of MSCs in prostate have been hampered by a lack of specific markers that permit both identification and linage tracing in vivo. Nestin is an intermediate filament protein described originally in neuronal stem cells during embryonic development and nerve injury 46. Recently, nestin^+^ cells have been shown to be a subpopulation of bone marrow derived MSCs and have the ability of self‐renewal and multilineage differentiation 34, 47, 48. Our study provides further evidence that nestin^+^ cells isolated from mice prostate have the classic MSC properties, including CFU‐F activity, fibrogenic, osteogenic, adipogenic and chondrogenic potentials. The main cell types in prostate stroma are smooth muscle cells and fibroblasts, both contributing to the synthesis of the extracellular matrix. Analysis of 6‐week‐old *Nestin‐Cre*, *Rosa26‐YFP^flox/+^* mice revealed that nestin^+^ lineage cells contribute significantly to prostate stromal formation physiologically including nearly 75% smooth muscle cells and 40% fibroblasts. Thus, nestin^+^ MSCs are involved in prostate growth and stromal tissue remodeling.

MSCs have both profibrotic and antifibrotic effects in various tissues and organs including liver, heart, lungs, skin, and pancreas. During the past few years, an increasing number of studies have evaluated the antifibrotic potential of MSCs. Conditioned media from MSCs attenuated fibroblast proliferation and collagen synthesis in vitro. In vivo, MSCs have been shown to attenuate fibrosis in nephrectomized rats 49 as well as having antifibrotic actions in animal models of cardiac disease 50. The propensity of MSCs migrate, proliferate and differentiate depending on the microenvironment surrounding them enhances their curative properties. The profibrotic role of MSCs finds usefulness in diabetic mice wound healing where aside producing ECM they contribute to wound healing by releasing various mediators 51. Profibrotic actions of MSCs in mouse model of liver damage have been demonstrated 52. MSCs migrated to sites during chronic rejection and differentiated toward a fibroblast phenotype in a rat model of chronic allograft heart rejection 53. In murine lungs fibroblast proliferation and matrix production were induced by MSC conditioned medium 54. In our study, we focused mainly on the recruitment of MSCs and their potential profibrotic role in BPH.

Bone marrow‐derived MSCs were recruited and then incorporated into the prostate epithelia through fusion during the process of prostate regrowth 43, 44. Peng et al. 55 demonstrated sonic hedgehog signaling from prostate basal epithelial cells to adjacent smooth muscle cells in mature prostate. They performed lineage tracing in the stroma to address smooth muscle cells as largely self‐sustained lineages during multiple rounds of castration‐induced involution and androgen‐mediated regeneration. Our parabiosis results demonstrated that nestin^+^ MSCs recruited from circulation differentiate to interstitial fibroblasts, but not to smooth muscle cells in prostate stroma. These results suggest there is heterogeneity of origin of interstitial fibroblasts and smooth muscle cells during prostatic hyperplasia.

TGF‐β signaling pathways are ubiquitous and fundamental regulators of cellular processes including proliferation, differentiation, migration, and survival, as well as physiological processes, including tissue development and wound healing 24. The appropriate regulation of these pathways is essential at all levels, especially at the ligand level, with either a deficiency or an excess of specific TGF‐β ligands resulting in human disease 31. The critical role of TGF‐β in prostatic stromal accumulation, which eventually leads to BPH, is well known 19, 20, 56, 57, 58, 59. Aberrant activation of TGF‐β canonical signaling has been observed in both rodent and human prostatic hyperplasia 18, 40. Recent studies showed that the murine pan‐specific TGF‐β neutralizing monoclonal antibody, 1D11, had the effect of pharmacological inhibition of TGF‐β in different mouse models, including osteogenesis imperfecta 60, chronic nephropathy 61, and asthma 30. In humans, fresolimumab, similar to TGF‐β antibody 1D11 in specificity, has been used for clinical trials in patients with idiopathic pulmonary fibrosis 62, primary focal segmental glomerulosclerosis 63 and cancer 64. Our study demonstrated that injection of 1D11 effectively reduced TGF‐β signaling, fibroblast numbers, and collagen deposition in prostatic hyperplasia mice with an accompanying reduction of the nestin^+^ cells in both prostatic stroma and blood. Knockout of Tgfbr2 in nestin^+^ cells attenuated the stromal accumulation in PE‐induced mice prostate. Clearly, upregulated TGF‐β signaling contributed to the higher number of nestin^+^ cells. The elevated levels of active TGF‐β mobilized MSCs from circulation to the stroma during the process of prostatic hyperplasia. Thus, targeting TGF‐β mediated recruitment of MSCs may represent a novel therapeutic approach for the treatment of prostatic hyperplasia.

## Conclusion

Activation of TGF‐β plays a role in the pathogenesis of benign prostatic hyperplasia. TGF‐β activation results in recruitment of MSCs and their subsequent differentiation into prostatic stromal cells. Thus, careful modulation of TGF‐β activity could provide potential therapeutic options in the management of benign prostatic hyperplasia.

## Author Contributions

L.W.: conception and design, collection and/or assembly of data, data analysis and interpretation, manuscript writing; L.X.: collection and/or assembly of data, data analysis and interpretation, manuscript writing; F.T.: data analysis and interpretation, manuscript writing; H.X., C.L., and Z.C.: collection and/or assembly of data, data analysis and interpretation; M.W.: manuscript writing; X.Z.: provision of study material or patients; L.Q.: provision of study material or patients, manuscript writing; X.C.: conception and design, financial support, administrative support, collection and/or assembly of data, data analysis and interpretation, manuscript writing, final approval of manuscript.

## Disclosure of Potential Conflicts of Interest

The authors indicated no potential conflicts of interest.
